# Report from the 18th International Symposium on Advances in Extraction Technologies (ExTech’2016) and the 22nd International Symposium on Separation Science (ISSS’2016), Toruń, 3–6 July 2016

**DOI:** 10.1007/s10337-016-3182-1

**Published:** 2016-10-22

**Authors:** Bogusław Buszewski

**Affiliations:** Chair of Environmental Chemistry and Bioanalytics, Faculty of Chemistry, Nicolaus Copernicus University, 7 Gagarin St., 87-100 Toruń, Poland

One might say that history repeats itself. The best confirmation of that fact is the 18th International Symposium on Advances in Extraction Technologies—ExTech’2016 and the 22nd International Symposium on Separation Science—ISSS’2016 which took place at the Culture and Congress Center in Toruń, 3–6 July 2016. One more time, over 350 specialists from 41 countries specialising in analytical chemistry, particularly the physicochemical methods of separation, met in the Gothic city of Toruń to summarise their achievements and to present new concepts both in the theory and practice of separation, enrichment, isolation and determination of the entire range of chemical substances. For the first time in history, the two great symposia ExTech and ISSS have been combined as one event. The former pertains to the methods of sample preparation based mainly on the extraction techniques. The latter deals with the physicochemical methods of separation and determination of organic and inorganic substances of different molecular mass and structure, both individual substances and their derivatives occurring in different matrices. The symposia were organised by the Committee of Analytical Chemistry of the Polish Academy of Sciences, Chair of Analytical Chemistry, Faculty of Chemistry of the Nicolaus Copernicus University and the Central European Group for Separation Science (CEGSS). The honorary patronage of this scientific event was assumed by the Minister of Science and Higher Education, Marshal of Kuyavian-Pomeranian Province, Mayor of Toruń, and His Magnificence Rector of Nicolaus Copernicus University in Toruń.

The program of the symposium was exceptionally rich. It included 49 plenary and panel lectures as well as 22 oral announcements mostly presented by young scientists. Moreover, during the four subject sessions, there were 258 poster presentations. An integral part of the conference were workshops and the exhibition of 17 manufacturers and distributors of equipment, reagents and accessories. During the symposium, particular attention was drawn to the accomplishments of the founders and pioneers of both extraction science (Prof. Walter Ernst) and chromatography (Prof. Mikhail Tswett). The first session was devoted to the theoretical achievements and fundamental aspects in the field of separation methods. It began with an insightful lecture by Prof. J. Pawliszyn (Canada) concerning the accomplishments and the future of sample preparation technology. Professor P. Jandera (Czech Republic), laureate of the European Tswett-Nerst award, presented the advances which have been made in multi-dimensional separation techniques. A new concept in characterising stationary phases and columns used in the so-called fast chromatography was presented by Prof. A. Felinger (Hungary). Professor O. J. Schmitz (Germany) presented new methodological solutions in the use of coupled separation techniques (LC–MS) for determination of biologically active substances.

Similarly, the other sessions brought numerous innovative and interesting solutions and proposals. They concerned such issues as new advances in sample preparation for the needs of biological and environmental analysis including analytics of food and natural products (Prof. E. Stashenko from Columbia, Prof. G. Mills from the UK, Prof. I. Vovk from Slovenia, Prof. D. Corradini from Italy or Prof. H. Jeleń from Poland). A particular emphasis was placed on the application of multi-dimensional systems and coupled and combined systems in the determination of biologically active substances on the cellular level (metabolism and omics) (Prof. H. Górecki from Canada, Prof. L. Mondello from Italy, Dr. J. Trafkowski from Germany, Prof. B. Bojko from Poland and Dr. P. Zuvela from South Korea) and monitoring changes which take place in the metabolic pathways and biogenic changes (Prof. M. Markuszewski and Dr. S. Studzińska and Dr. K. Skalicka Woźniak from Poland, Prof. A. Maruśka from Lithuania, Dr. W. Filipiak and Prof. P. Mochalski from Austria).

The research paper speeches concerned miniaturisation and robotisation both in the sample preparation and determination of analytes on the sub-ultra traces (Prof. S. Fanali from Italy, Prof. V. Pichon and Dr. D. Thiebaut from France, Prof. L. Chimuka from RSA, Prof. J. Nogueira from Portugal and Prof. G. Ouyang from China). These subjects well correspond with the issues connected with developing new generation of materials such as packings, analytical columns and columns used in micro- or nano-scale (Prof. Z. Jang from China, Prof. E. Psillakis from Greece, Dr. L. Pelit from Turkey, Dr. M. Szumski from Poland and Prof. A. Malik and J. Anderson from the USA). The so-called chip laboratory (pill-lab) is a challenge not only for researchers studying miniaturisation and robotisation of the combined process of sample preparation and final determination (Prof. M. Kaljurand from Estonia or Dr. Vaitz from Germany). This issue was superbly presented in the research paper presented by Prof. Z. Brzóska (Poland), who pointed out the possibilities of using these solutions in modern analytics.

The solutions aimed at the application of these achievements for the determination of toxic substances of endo- and exogenic origin or xenobiotics in different matrices, particularly in water, were presented by Prof. H. Lee (Singapore), Prof. M. Bruzzonitti (Italy), Prof. D. Knapp (Germany), Dr. V. Comann (Romania) or Dr. M. Gerstiuk (Ukraine), in cosmetics (Prof. M. Llompart from Spain) or monitoring of technological processes (Prof. E. Rosenberg from Austria). All this was perfectly complemented by works devoted to the theoretical considerations containing description of separation mechanisms presented by: Prof. S. Pedersen-Bjergaard from Norway, Prof. I. Malinowska (Poland), Dr. T. Edge (UK), Prof. H. Bagheri (Iran), Dr. J. Weiss from Germany, Dr. D. Berek from Slovakia and Prof. J. Koziel (USA).

The presented subject matter was also well suited by short oral announcements made by young researchers (20 papers) and research news presented in the form of posters (258). The latter were also accompanied by short oral presentation in front of a panel of judges headed by Prof. T. Górecki. The jury evaluated the scientific innovation, the form and manner of the presentation. The winners/laureates of the contest were:

- **J. Mazina**, A. Tretjakova, P. Saar-Reismaa, J. Gorbatsova, M. Kulp, M. Vaher, E. Erme & M. Kaljurand, Technical University, Tallin (Estonia), poster S1-P71,
- **M. Gładysz**, M. Woźniakiewicz, P.M. Nowak & P. Kościelniak, UJ Kraków (Poland), poster S1-P34,
- **T. Hajek**, P. Jandera & M. Stankova, University of Pardubice (Czech Rep.), poster S2-P17,
- **M. Skoczylas**, Sz. Bocian & B. Buszewski, UMK Toruń (Poland), poster S2-P35,
- **M. Matczuk**, J. Legat & M. Jarosz, Politechnika Warszawska (Poland), poster S3-P17,
- **C. Molins-Legua**, P. Camping-Falco, Y. Moliner-Martinez, J. Vordu-Andres, J. Pla-Tolos & M. Munnos-Ortuno, University of Valencia (Spain), poster S3-P19,
- **E. Gijonfriddo**, E.A. Souza-Silva, S. De Grazia, X. Li & J. Pawliszyn, University of Waterloo (Canada), poster S4-P17,
- **L. Rivoira**, S. Studzińska, M. Szultka-Młyńska, M. C. Bruzzonitti, M. Ligor & B. Buszewski, University of Torino (Italy)/UMK(NCU) Toruń (Poland), poster S4-P33.

The material and book prizes were funded by: Springer, Wiley–VCH, Polygen company, Polish Chemical Society and the Committee of Chemical Analytics of the Polish Academy of Sciences.

The jury also awarded two prizes for the oral presentations of young researchers:

- **M. Buszewska-Forajta** & R. Kaliszan, Gdański Medical University (Poland), OP-7,
- **G. A. Gomez-Rios**, N. Reyes-Garces, E. Boyaci & J. Pawliszyn, Waterloo University (Canada), OP-12.

The prizes in the form of the covered registration fee at the next conferences of ExTech 2017 (Santiago di Compostella, Spain) and ISSS 2017 (Vienna, Austria) were sponsored by the organising committees of these symposia. Moreover, the organising committee of ITP 2017 in Gdańsk awarded a prize to L. Rivoira (Turin, Italy) by financing the participation in this conference.

During ExTech 2016 & ISSS 2016, apart from the up-to-date and abundant scientific program, the organisers provided the participants with an extensive and attractive cultural offer and the presentation of the traditions of the Kuyavian-Pomeranian region and the city of Toruń. The opening concert by Sławek Wiercholski and Nocna Zmiana Bluesa band at the Jordanki Congress and Culture Center does not require any comment. It was an excellent prelude to the working sessions of the symposium. Similarly, the performance given by the Culture Center from Świecie presenting the folk dance, songs and customs of Poland and the region in the setting of the open-air Ethnographic Museum showed the richness and diversity of Polish culture.

The show also included a performance by a traditional gypsy band as well as young musical and vocal talents during a common feast, which warmed up the atmosphere of the meeting. Similarly, the participants enthusiastically received the performance of the Toruń singer M. Lubomski and his band given after the official gala dinner at Jordanki Congress and Culture Center. The participants thoroughly enjoyed the delicious dishes served by Gołębiewscy catering company and the excellent music by Rumińscy band. An important element of the program was the post-conference sightseeing tour of Toruń Along the Gothic Trail including a visit to the Live Museum of Gingerbreads. Despite the rainy weather, the participants enjoyed the tour which was confirmed by numerous letters and expressions of thanks for the organisation and the warm familiar atmosphere. This gives us enormous satisfaction of the well-done important tasks that we were entrusted with and makes us committed to live up to the expectations of the future.

All this would not have been possible without the invaluable financial support of the government institutions, local and regional authorities and commercial companies (Ministry of Science and Higher Education, Marshal of Kuyavian-Pomeranian region, Mayor of Toruń, Polish Academy of Sciences, companies such as: LECO-Polska, Perlan Technologies, Shim-Pol, AlChem, VWR, TZMO, La Rive and many others. The organisers and the participants express their gratitude for this support on numerous occasions.

Prof. Dr. Bogusław Buszewski, DSc.

Chairman of the Scientific and Organising Committee

Toruń, 15 July 2016

ExTech’2016 & ISSS’2016

